# Ocular Manifestations of Systemic Lupus Erythematosus: A Review of the Literature

**DOI:** 10.1155/2012/290898

**Published:** 2012-07-02

**Authors:** Neal V. Palejwala, Harpreet S. Walia, Steven Yeh

**Affiliations:** Section of Vitreoretinal Disease and Surgery, Department of Ophthalmology, Emory Eye Center, Emory University School of Medicine, Atlanta, 30322 GA, USA

## Abstract

About one-third of patients suffering from systemic lupus erythematosus have ocular manifestations. The most common manifestation is keratoconjunctivitis sicca. The most vision threatening are retinal vasculitis and optic neuritis/neuropathy. Prompt diagnosis and treatment of eye disease is paramount as they are often associated with high levels of systemic inflammation and end-organ damage. Initial management with high-dose oral or IV corticosteroids is often necessary. Multiple “steroid-sparing” treatment options exist with the most recently studied being biologic agents.

## 1. Introduction

Systemic lupus erythematosus (SLE) is a chronic, autoimmune, connective tissue disorder affecting multiple organ systems often with a relapsing and remitting clinical course. Prevalence, clinical manifestations, and morbidity vary significantly between the developing and industrialized worlds. While SLE is more common in people of African and Asian descent, thrombotic complications are more common in Caucasian patients [1]. The highest prevalence has been reported in Italy, Spain, Martinique, and the UK Afro-Caribbean population [2]. The median age of onset is between the late teens and early 40s with a 9 times higher incidence in women compared to men. Ocular manifestations—occurring in up to one third of patients—can be associated with significant morbidity and also a marker for overall systemic disease activity.

## 2. Genetic Considerations

Concordance rates for SLE among monozygotic and dizygotic twins are 25% and 2%, respectively, suggesting a significant genetic contribution [3]. Major histocompatibility complex genes, such as HLA-A1, B8, and DR3 [4], as well as alleles that cause deficiency in complement components—C1q, C2, and C4 [5]—have all been linked to lupus.

## 3. Mechanism of Disease

SLE is a complex disease process demonstrating dysregulation of the immune system at multiple levels. Autoantibodies against double-stranded DNA were first isolated from kidney specimens in patients with lupus nephritis in 1967 [6]. Other autoantibodies that have been implicated in disease include anti-Ro, La, Sm, nucleosome, NMDA receptor, phospholipid, and *α*-actinin. Two major theories exist on how these autoantibodies cause tissue damage. The first model suggests that anti-double-stranded DNA antibodies bind to circulating nucleosomes to form immune complexes that then get deposited in end-organ capillary beds such as the renal glomerulus and activate immune/inflammatory responses [7]. The second hypothesizes that these autoantibodies cross-react with normal renal proteins causing tissue destruction [8]. The source of autoantigens that trigger the formation of the aforementioned antibodies is thought to arise from apoptotic cells. Normally, early complement factors, such as C1q, bind cellular debris from apoptotic cells, which facilitate phagocytosis by macrophages. Deficiency of such complement factors is an independent risk factor for the development of SLE [5].

Mass production of autoantibodies relies on multiple factor, which have each independently been targeted as potential immunotherapy in the treatment of lupus. Important steps include T-cell activation via antigen binding to the T-cell receptor and proper costimulation; T-cell activation of B cells; production of cytokines such as TNF-*α*, INF-*γ*, IL-10, and B-lymphocyte stimulator. 

Medications, hormonal influences, and other factors such as sunlight have all been implicated in disease exacerbation. Drug-induced lupus, most commonly due to procainamide, hydralazine, and quinidine, usually presents with disease involving the skin and joints with renal and CNS manifestations being much more rare [9]. Hormonal replacement therapy has been associated with an increased risk of mild-to-moderate flares [10]. 

## 4. Diagnostic Criteria

According to the 1982 revised criteria for systemic lupus erythematosus, a diagnosis of SLE can be made by the serial or simultaneous presentation of at least 4 of the following 11 criteria: malar rash, discoid rash, photosensitivity, oral ulcers, nonerosive arthritis, serositis, renal dysfunction, neurological derangements (i.e., seizures or psychosis), hematologic disorder (i.e., anemia, leukopenia, thrombocytopenia), immunologic disorder (i.e., anti-DNA antibody, anti-Sm antibody, and false positive VDRL testing), and presence of antinuclear antibodies. 

## 5. Ocular Manifestations

SLE can affect the periorbita, ocular adnexa, eye, and optic nerve. The most common association is keratoconjunctivitis sicca, while the most visually devastating sequelae occur secondary to optic nerve involvement and retinal vaso-occlusion. 

### 5.1. Orbit

Orbital involvement is a rare manifestation of SLE. Vasculitis, myositis, and panniculitis have all been described. Signs and symptoms include proptosis, enophthalmos, orbital pain, blurred vision, chemosis, and restriction of extraocular movements. 

Orbital vasculitis leads to nonperfusion of the globe and extraocular muscles. This has been shown to cause irreversible vision loss secondary to ischemic injury to the optic nerve as well as elevated intraocular pressure from neovascular glaucoma [11].

Orbital myositis is often initially misdiagnosed as bacterial orbital cellulitis, as it usually presents with significant pain, proptosis, periorbital swelling, and globe restriction. CT and orbital ultrasound are both valuable in demonstrating enlargement of one or multiple extraocular muscles. Creatinine kinase, aldolase, and myoglobin levels are markedly elevated. Inflammation and symptoms typically respond to steroids [12, 13].

Subcutaneous inflammation secondary to SLE was first described by Kaposi in 1883, and the term “lupus erythematosus panniculitis” was coined in 1940 [14]. It is most commonly encountered in the setting of discoid lupus erythematosus. Clinical findings include tender deep subcutaneous nodules usually involving the proximal extremities, trunk, face, and scalp [15]. Orbital involvement is very rare and has only been reported in a handful of paper. Histopathology shows perivascular lymphocytic infiltration [16]. Response to steroids can be quite dramatic in most cases [16–18]; however, few cases have shown a more virulent course with significant enophthalmos secondary to fat atrophy [19] and even melting of orbital structures [20].

### 5.2. Periorbita

Periorbital edema is an uncommon manifestation of systemic and discoid lupus erythematosus with an overall incidence of 4.8% [21]. It is most common in patients of African decent [22]. Violaceous swelling with overlying eczematous changes without any skin necrosis is seen. Some cases can resemble chronic blepharitis [23]. Etiologies include nephrosis, increased vascular permeability, dermal mucin deposits, and angioedema secondary to C1 deficiency. Treatment options include observation [23], topical/intradermal/systemic corticosteroids [24], and antimalarials [23].

### 5.3. Eyelids

Typical lesions of discoid lupus erythematosus are slightly raised, scaly, and atrophic. Most commonly, they occur on the head, face, neck, and other sun-exposed areas. Rarely does it affect the eyelids. Histopathologic study shows a hyperkeratotic epithelium with liquefactive degeneration of the basal layer and a dense perivascular/periappendageal lymphocytic infiltration [25, 26]. Diagnosis in most cases is delayed because lesions are often mistaken for blepharitis and eczema. Patients most commonly present with chronic erythema, blepharoconjunctivitis with inflammation of the meibomian glands. Long-term complications include madarosis, lid scarring, and cicatricial ectropion/entropion [26, 27].

### 5.4. Ocular Surface

The most common ocular manifestation of SLE is keratoconjunctivitis sicca with the majority of patients endorsing at least one dry eye symptom [28]. Dryness can occur from multiple etiologies. Most patients with SLE develop a secondary Sjogren's syndrome. In their review of 283 SLE patients, Manoussakis et al. [29] identified 9.2% who had developed Sjogren's syndrome (SS). The SLE-SS group had a higher frequency of Raynaud's phenomenon, anti-Ro antibody, anti-La antibody, and rheumatoid factor and a lower frequency of renal involvement, lymphadenopathy, and thrombocytopenia. These patients tend to undergo a more benign course with a significantly reduced mortality and need for immunosuppression [30]. The hallmark of disease is a decreased production of the aqueous layer of the tear film.

An abundance of proinflammatory markers such as IL-17 [31, 32] can be found in the tear film of SLE patients. These are some of the same markers that are found in cicatrizing inflammatory conditions such as Steven Johnsons syndrome. Clinical findings can include symblepharon formation, forniceal foreshortening, and exposure keratopathy. Histopathological findings include loss of goblet cells, keratinization of the conjunctival epithelium, monocellular infiltration, and granuloma formation in the substantia propria [32]. Immunopathology shows immune complex deposition within the epithelial basement membrane with an increased number of CD4+ and CD8+ T cells, B cells, and macrophages [32, 33].

### 5.5. Episclera/Sclera

Episcleritis is generally a benign inflammation of the episclera. Typically occurring in young women, symptoms include a dull ache, red eye, and tearing. Decreased visual acuity and severe pain are uncommon. Systemic associations are extremely rare in adults, and a systemic workup is not necessary. Incidence in adult patients with SLE has been reported at 2.4% [34]. However, in children, episcleritis is much more rare but systemic associates are much more common. Read et al. [35] found 6 of 9 patients in their series on pediatric episcleritis to have systemic connective tissue disease. Treatment options include observation or topical/systemic nonsteroidal anti-inflammatory drugs.

**Figure 1:**
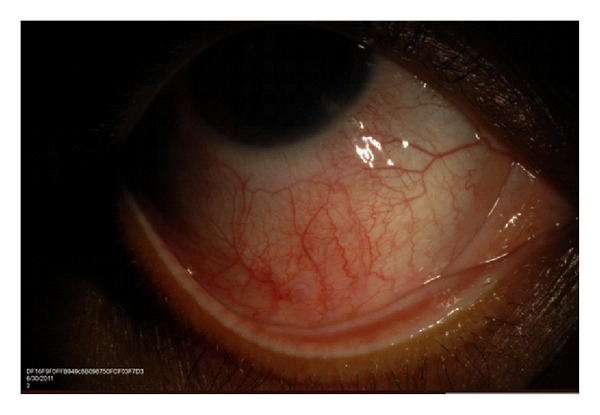
Slit-lamp photo demonstrating diffuse anterior scleritis in a patient with SLE.

Scleritis is a more painful and potentially a vision-threatening condition that warrants prompt treatment. Anterior scleritis can be nodular or diffuse and presents with a red, painful eye that is tender to touch. The injected deep episcleral vessels give a violaceous due to the sclera, which is best appreciated in natural light (Figure 1). Posterior scleritis on the other hand may not be associated with a red eye because it affects sclera posterior to the equator of the globe. Presenting symptoms are pain, blurred vision, limited eye movements, and proptosis. Blurred vision is most commonly caused by exudative retinal detachment, macular distortion due to a large scleral mass, and cystoid macular edema.

### 5.6. Cornea

Corneal epitheliopathy, scarring, ulceration, and filamentary keratitis can all occur secondary to keratoconjunctivitis sicca. More rare corneal complications include peripheral ulcerative keratitis [36], which can be a marker of active systemic vasculitis, interstitial keratitis, and keratoendothelitis [37]. Spectral microscopy has been used to show dysfunctional appearing corneal endothelial cells in both patients with corneal edema and asymptomatic patients [38].

Corneal biomechanical properties differ in SLE. Yazici et al. [39] used Reichert ocular response analyzer measurements to show that corneal hysteresis and corneal resistance factor were both lower in SLE patients which can lead to an underestimated IOP and development of keratoconus [40].

### 5.7. Retina

Lupus retinopathy is one of the most common vision-threatening complications of systemic lupus erythematosus with an incidence of up to 29% in patients with active systemic disease. A strong correlation exists between presence of retinopathy and CNS disease [41]. The most common pattern of retinopathy is microangiopathy similar to diabetic and hypertensive retinopathy. The earliest findings are small intraretinal hemorrhages and cotton wool spots [42]. Pathogenesis is attributed to deposition of immune complexes in the vessel wall and microemboli. Histopathology shows immunoglobulin and complement deposits, perivascular monocellular infiltrate, and rarely fibrinoid necrosis [43, 44]. Studies using fluorescein angiography describe hyperpermeability of arterioles and venules as well as capillary nonperfusion [45]. Although it is poor prognostic factor for survival, visual outcomes in this group are usually very good [46].

Retinal vasculitis, a subset of retinal vasculopathy featuring inflammation of the retinal arterioles or venules, tends to have poorer visual outcomes and present in an acute onset fashion. A large percentage of these patients have concomitant antiphospholipid antibodies including anticardiolipin and lupus anticoagulant. In one study, 77% of patients with SLE and retinal involvement had positive antiphospholipid antibody titers, whereas only 29% of SLE patients without retinal disease had positive titers [47]. Histopathologic specimens show fibrinoid change with thrombus formation without a true arteritis [48]. CNS vascular disease demonstrates similar pathology, thus providing a link between CNS vasculitis and severe lupus vasculopathy [49]. In 1984, Hall et al. [50] first reported the link between severe lupus retinal vasculopathy and presence of antiphospholipid antibodies. Since that time, multiple cases have been demonstrating severe vision loss secondary to central retinal artery/vein occlusions, vitreous hemorrhage, retinal ischemia, and neovascularization [47–53]. While the milder form of retinal vasculopathy is mediated by immune-complex deposition and inflammation, the more severe vaso-occlusive disease stems from fibrinoid degeneration/necrosis without significant inflammation. 

**Figure 2:**
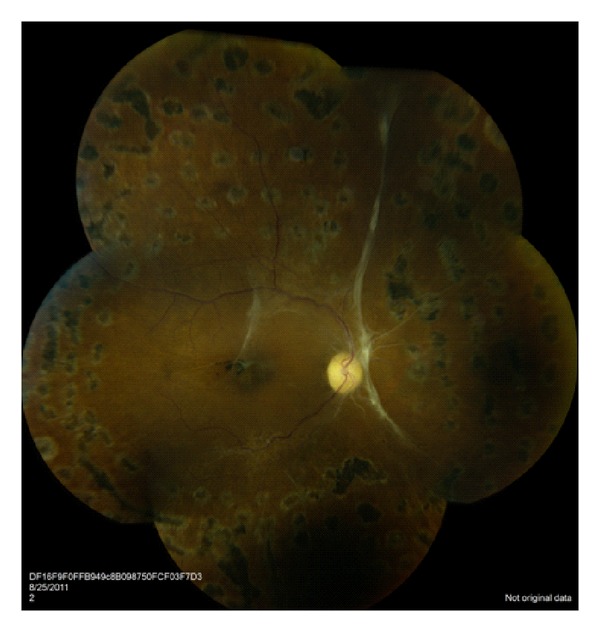
Fundus photograph demonstrating severe retinal vasculitis. Significant ischemia is present which is highlighted by the attenuated and sclerotic vasculature. Panretinal photocoagulation was required to treat ischemic and neovascular complications.

Immunosuppression has been successful in improving the appearance of the retinopathy; however, visual recovery has only been reported in few cases. The permanent loss of visual acuity is likely due to retinal ischemia. Addition of anticoagulation to immunosuppression helps to stabilize retinal disease and prevent further vascular events [48]. Other therapies that have been reported for severe disease include plasmapheresis [54] and plasma exchange [55]. Panretinal photocoagulation, intravitreal antivascular endothelial growth factor agents, and vitrectomy may also be considered for the treatment of complications of ocular ischemia (Figure 2). 

### 5.8. Choroid

**Figure 3:**
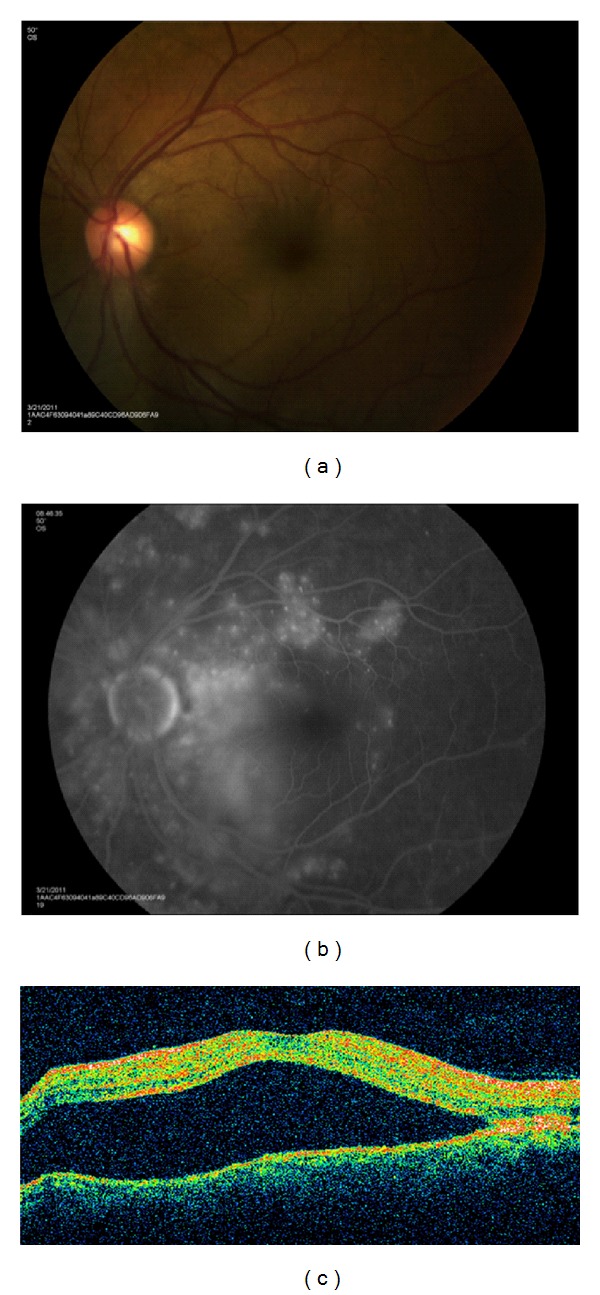
(a) Macular serous retinal detachment in a patient with lupus choroidopathy. (b) Multiple areas of hyperfluorescence seen on fluorescein angiography caused by increased vascular permeability of the choroidal circulation. (c) Large accumulation of subretinal fluid is seen on optical coherence tomography.

Lupus choroidopathy with exudative retinal detachments is a rare ocular manifestation with fewer than 40 patients reported in the literature (Figure 3). It is generally seen in patients who have highly active disease including CNS vasculitis and nephropathy as well as uncontrolled blood pressure. Clinical diagnostic ophthalmic imaging is paramount for the diagnosis of choroidal and retinal pathologies. Specifically indocyanine green is extremely valuable for evaluating choroidal vascular and tissue inflammation, while fluorescein angiography is helpful in identifying optic nerve inflammation, retinal vascular disease, retinal ischemia, and macular edema. Baglio et al. [56] used indocyanine green angiography (ICG) to demonstrate that subtle changes in the choroidal circulation can be seen in patients with SLE-associated nephropathy, while similar findings are not seen in SLE patients without renal involvement. The pathogenesis is thought to be multifactorial; uncontrolled hypertension [57], immune complex deposition in the choriocapillaris [58], and antiretinal pigment epithelium antibodies [59] have all been implicated as contributing factors. 

Recently, ICG imaging has been used to visualize the choroidal circulation in lupus choroidopathy. Studies have shown focal, transient early-phase hypofluorescence followed by late-phase diffuse hyperfluorescence, distortion of the large choroidal vessels, and also focal clusters of choroidal hyperfluorescence in the intermediate phase. Transient early hypofluorescence and late hyperfluorescence are likely secondary to choroidal vascular perfusion delay with subsequent leakage due to an increase in vascular permeability, which are also observed in other vascular and inflammatory diseases. Unique findings include focal areas of hyperfluorescence in the intermediate frames, which may represent ICG staining of immune complexes [60]. 

Although it is a marker of high disease activity, lupus choroidopathy has been shown to be responsive to corticosteroids and other forms of immunosuppression. Given its associations with CNS and renal disease, the presence of choroidopathy is likely an indication for aggressive, long-term immunosuppression. 

### 5.9. Optic Nerve/Central Nervous System

Optic nerve disease is a rare manifestation of SLE and consists of optic neuritis and ischemic optic neuropathy [61]. Presenting visual acuity in SLE-associated optic neuritis is poor with most patients seeing worse than 20/200 [62]. In the Optic Neuritis Treatment Trial (ONTT), only 35.9% had a similar vision [63]. Visual recovery is variable in most patients and can range anywhere from full recovery to count fingers vision. In a study by Lin et al. [62] only 50% of patients recovered to better than 20/25, while 37.5% maintained a visual acuity worse than 20/200. In ONTT, 87% of patients recovered to better than 20/25 at 5 years of followup [63]. The increased severity of disease in SLE-associated optic neuritis compared to idiopathic optic neuritis stems from differences in pathogenesis. SLE-optic neuritis is not due to a primary inflammatory demyelinating process but rather an ischemic process that can cause subsequent demyelination and axonal necrosis. The degree of axonal loss correlates to visual outcome [64]. Luckily, the optic neuritis responds dramatically to corticosteroid treatment [65]. Early diagnosis and prompt treatment with high-dose corticosteroids is associated with better visual outcomes [62]. 

The neuromyelitis optica spectrum disorders (NMOSDs) are characterized by a combination of optic neuritis and transverse myelitis. Few cases have been reported in the literature of the presentation of NMOSD in SLE [66–68]. A recent paper by Jarius et al. [69] demonstrates a high association of aquaporin-4 antibodies in patients with connective tissue disease and symptoms suggestive of NMOSD. The antibodies cause tissue destruction by complement activation. Aquaporin-4 antibody positivity has important clinical implications as it is associated with a relapsing course of myelitis and optic neuritis and can lead to blindness and immobility quickly if not treated [70]

Optic neuropathy in SLE is caused by an ischemic process that affects the small vessels supplying both the optic nerve head and retrobulbar nerve. It usually presents as an acute loss of vision with an altitudinal visual field defect with or without optic disc edema. The disease is most commonly bilateral except in patients with circulating antiphospholipid antibodies. In this subset, a focal thrombotic event in the ciliary vasculature is thought to occur as opposed to a generalized vasculitis [71]. Standard treatment for lupus optic neuropathy includes intravenous high-dose corticosteroids followed by an extended oral taper [72]. Other studies have shown success with other immunosuppressive agents such as cyclophosphamide, cyclosporine, methotrexate, and azathioprine [73, 74].

Eye movement abnormalities are common in SLE and have been reported in up to 29% of patients [75]. Ischemic microvascular disease of the brainstem is usually the etiology. Sixth nerve palsies are the most common cause of disconjugate gaze abnormalities [75], while internuclear ophthalmoplegia is the most common cause of conjugate gaze abnormalities [76–78]. 

Retrochiasmal involvement can cause visual hallucinations, visual field defects, nystagmus, and cortical blindness. Few cases of idiopathic intracranial hypertension have been reported. 60% of cases reported in the literature are associated with antiphospholipid antibodies [79]. 

## 6. Therapeutic Considerations

Treatment options for SLE range from nonsteroidal anti-inflammatory drugs, corticosteroids, antimalarials, immunomodulatory, and biologic agents. Significant ocular involvement—orbital inflammation, scleritis, retinal vasculitis, choroiditis, and optic neuritis—warrants systemic therapy. The goal of treatment is to suppress immune activity, specifically decreasing the level of autoantibodies. 

Corticosteroids are the mainstay and most effective short-term therapy for SLE [80]. They inhibit both the innate and adaptive immune response by preventing proliferation and inducing apoptosis of T cells, B cells, and macrophages as well as reducing levels of cytokines and prostaglandins [81, 82]. Generally, by the time patients present with ocular manifestation, they have a high level of systemic inflammation. Previous papers have shown that a high correlation exists between CNS vasculitis and retinal vasculitis [49] as well as nephritis and choroiditis [56]. Nguyen et al. recently described a series of four of 28 patients with choroidopathy who died from lupus-related complications [83]. Early and aggressive treatment is needed for this group to prevent increased morbidity and mortality. Thus, it is of extreme importance that patients presenting with severe ocular manifestations be treated with high-dose oral or even IV steroids early on in the disease course. Periocular steroid injections may have a role in unilateral/asymmetric disease; however, they should be used cautiously and avoided in patients with scleritis. 

Steroid-sparing immunosuppressive agents are used in a large amount of patients secondary to treatment failure or harmful side effects of corticosteroids. Antimalarials such as chloroquine and more commonly hydroxychloroquine are often used. These medications are highly efficacious in curtailing future flares with fewer side effects compared to other immunomodulatory drugs such as alkylating agents. However, ocular effects of these drugs are well known. Irreversible vision loss secondary to a drug-induced maculopathy has been well documented in the literature. Factors associated with high risk of developing maculopathy include greater than 5–7 years of therapy, greater than a cumulative dose of 1000 g of hydroxychloroquine, impairment of liver or kidney function, obesity, age greater than 65, and preexisting retinopathy. The American Academy of Ophthalmology recommends a baseline-dilated eye exam on all patients starting hydroxychloroquine followed by annual exams starting at 5 years after initiating therapy. A Humphrey 10–2 automated visual field test along with multi-focal electroretinogram, spectral domain optical coherence tomography, or fundus autofluorescence should be performed at each of these visits. Discontinuation of the drug should be recommended at the earliest sign of toxicity [84]. Unfortunately, cases of progression of retinopathy despite cessation of therapy have been reported [85].

Methotrexate, azathioprine, mycophenolate mofetil, cyclosporine A, cyclophosphamide, and chlorambucil have all been employed with varying degrees of success. 

In the past few years, newer drugs, categorized as biological agents, have emerged targeting specific molecules involved in B- and T-cell activation. One of the first to be utilized in SLE was rituximab, a chimeric murine/human anti-CD20 antibody. Multiple studies have shown clinical improvement in refractory patients [86, 87]. Rituximab has also shown efficacy in treating noninfectious forms of ocular inflammation including that secondary to SLE [88].

## 7. Conclusions

In summary, a myriad of ocular manifestations of systemic lupus erythematosus have been described, and in some patients, these findings may be a presenting sign of systemic disease. Moreover, their presence can be a sign or a marker of disease activity. In the cases of choroidopathy and retinopathy, ophthalmic findings can be a poor prognostic systemic risk factor with the potential for both ophthalmic and systemic morbidity. For this reason, treatment typically involves a considered assessment of both the systemic and ophthalmic findings in determining the proper therapy and duration of treatment. Close communication between the consultant ophthalmologist and treating rheumatologist is critical in the effective management of these complex clinical situations.
